# 

**Published:** 1893-08-12

**Authors:** 


**OUR NEW OFFICES**

428, Strand, W.C., will henceforth be the Office of this Journal.

**Notices of Births, Deaths, and Marriages,** are inserted in this column at a charge of 2s. 6d. for an announcement not exceeding four lines.

### NOTICE TO CORRESPONDENTS.

All MS., Letters, Books for Review, and other matters intended for the Editor should be addressed THE EDITOR, THE LODGE, PORCHESTER SQUARE, LONDON, W.

The Editor cannot undertake to return rejected MS., even when accompanied by stamped directed envelope.

**Notice to Advertisers and Subscribers.**

All Advertisements, Orders for Copies of THE HOSPITAL, and Business Communications should be addressed to The Publisher, *not to the Editor*, THE HOSPITAL, 428, Strand, W.C.
The Cost of Subscription, post free, is as follows:—Per week, 2½d.; per quarter, 2s. 6d.; per half-year, 5s.; per annum, 8s. 6d. Foreign:—Per annum, 10s. 8d.; payable, in all cases, in advance.

**"Burdett's Hospital Annual," 1893.**

Fourth year of publication. 700 pp. Boards, gilt lettering. A most complete guide to British, Colonial, Indian, and American Hospitals, Dispensaries, Nursing and Convalescent Institutions, and Asylums. Price 5s. Published by THE SCIENTIFIC PRESS (Limited), 428, Strand, W.C.

**NOTICE.—The Index for Vol. XIII. (ending March 1893) is on sale at the Office of this Journal, price 2½d., post free.**

### SCRAPS AND GLEANINGS.

The cholera has broken out in Naples and Alexandria.

The late Mr. Herbert H. Keeling, of Eltham, has bequeathed £100 to the Eltham Cottage Hospital.

The London Hospital has received a gift of £500 towards the fourth quinquennial fund of the hospital.

Attempts are being made at Cirencester to establish a Hospital for Infectious Diseases in that vicinity.

At a cost of £100,000, Glasgow is providing new sewage precipitation works for the eastern division of the city.

The London County Council have agreed to purchase a large site near Bexley for the erection of a new pauper lunatic asylum.

Mr. W. F. Low, of Wimpole Street, has bequeathed £100 each to the London Fever Hospital and University College Hospital.

The Duke of Westminster has sent a donation of £500 towards the funds of the Metropolitan Hospital, Kingsland Road, N.E.

A carnival in aid of local charities was held in Edinburgh last week. The Lord Mayor of Dublin drove in state to the scene of the festivities.

At a recent meeting of the Council of the Royal College of Surgeons a resolution was put forward advocating separate meetings for fellows and members.

The total revenue of the Arbroath Infirmary for the year was £802, and the Infirmary ordinary expenditure £761, showing a balance on the account of £41.

The Women's Printing Society shows increasing prosperity. The last annual report states that 5 per cent. is paid on the share capital and bonuses to workers besides.

The Metropolitan Asylums Board at a fortnightly meeting agreed that the recently-acquired site at Lewisham should be called the Brook Hospital for infectious disease.

The quarterly statement of accounts for the Bedford General Infirmary showed a gain of four guineas. Seven patients had been treated during that period in the feverhospital.

Lady Stamford sent a donation of £25 to the Leicester Infirmary with a request that it should be spent amongst the patients as a small commemoration of the recent Royal wedding.

It will be a matter of astonishment to those inclined prosaically to believe in definite scientific methods, to learn that the Worthing authorities have applied to a "water diviner" to find new water springs.

A legacy of £1,000 will be added to the funds of the Arbroath Convalescent Home in the course of the present year. The endowment fund of the infirmary is increasing, and has now reached about £10,220.

The new central block which was opened recently by the Princess Christian in connection with the North London Hospital for Consumption, provides accommodation for nurses and for 35 extra patients.

The table showing the various defects found in the children of the poor attending eighteen schools is published in the *British Medical Journal*. The table is interesting, and the statistics will cause surprise to many.

The Leathersellers' Company have sent £100 in aid of the establishment of the Northern Polytechnic Institute, which is designed for the technical instruction and physical recreation of the overcrowded district of London north.

The Secretary of the Royal Hospital, Richmond, has received from the Kew Light Opera Company, as a result of the "Mikado" performances in May last, a cheque for £28; this sum has been appropriated to the Princess May Ward Fund.

The Leicester Amateur Dramatic Club also have benefited the institution with a further donation of £26, accompanied by the request that a cot should be named to them, which they would endeavour to support through proceeds from their entertainments during the year.

At the meeting of the Edinburgh Town Council on July 11th, the Public Health Committee recommended general approval of a block plan of the proposed additions to alterations on the City Hospital, and asked a remit to prepare plans and specifications for the same.

The Leicester Sanitary Committee, it is reassuring to see, have made the first step in the right direction, having at length announced that they are desirous of receiving offers of land to be let on lease, with option of purchase, as a site for a contageous diseases hospital.

The Manchester Unity of Oddfellows are taking steps in various parts of the country to establish convalescent homes. The first of these will shortly be opened in the Birmingham district, this great city having been chosen as the base from which operations will be worked.

At the last meeting of the London Asylums Board, statistics were supplied to show a decrease of the number of small-pox cases. The number during the fortnight having gone down from 397 to 353, further statements went to prove that the danger of a serious epidemic is now over.

During the three years since the establishment of the Surrey Convalescent Home at Seaford, 600 patients having received benefits through the gift of an anonymous donor. Founders' Day, July 24th, was commemorated by a luncheon in the dining-hall, at which Sir Trevor Lawrence presided.

The numerously-attended meeting held at Grosvenor House to advocate the claims of the King's College Hospital not only received a generous precedent through the gift of the Duke of Westminster, but a further £500 was contributed by Mr. Twining, which with other smaller sums made a total of £2,000.

The invested funds for the Royal Isle of Wight Infirmary produce only £745, inclusive of the partial endowment of the Convalescent Home appertaining to it. The general expenditure for the year exceeds £2,500; therefore, the establishment is mainly dependent on contributions, which appear to be falling in but slowly.

The University of Pennsylvania is shortly to possess a fine new laboratory. This will contain rooms adapted to experiments of every kind. The heating and ventilating will be of the last designs, whilst a special apparatus for carrying off nauseous smells will be supplied. An amphitheatre for lectures, and apartments for quantitative analysis will be also designed.

### Medical Vacancies and Appointments.

### APPOINTMENTS.

W. Arnold, appointed Medical Officer for the Great Milton and Little Milton District of the Thame Union. H. M. Bowman, M.D.Lond., M.R.C.P., appointed Assistant Physician to the Royal Hospital for Diseases of the Chest, City Road. J. Buck, L.R.C.P., L.R.C.S.Edin., appointed Medical Officer of Health for the Rural Sanitary District of the Hunslet Union. W. K. Bullmore, M.D.St.And., M.R.C.S.Eng., reappointed Medical Officer of Health for the Falmouth Urban Sanitary District. C. H. Cribb, B.Sc.Lond., F.I.C., reappointed Public Analyst for the Strand District. H. Cropley, F.R.C.S.Eng., appointed Medical Officer of Health for the Kingsthorpe Urban Sanitary District of the Northampton Union. P. E. Davies, L.R.C.P.Lond., M.R.C.S.Eng., appointed House Surgeon to the Kidderminster Infirmary. G. H. Doman, B.A., M.B., B.C.Cantab., appointed Certifying Surgeon under the Factory Act for Westhoughton; and Medical Officer and Public Vaccinator for Westhoughton District of the Bolton Union. W. J. H. Fletcher, L.R.C.P., M.R.C.S.Edin., appointed Medical Officer for the Uttoxeter and Leigh Districts and Workhouse of Uttoxeter Union. A. W. German, appointed Assistant Medical Officer for the Mile Road Infirmary of the West Derby Union. A. Harris, M.B., C.M.Edin., appointed Medical Officer of Health for the Thetford Urban Sanitary District. F. D. Holmes, B.A.R.U.I., L.R.C.P.Edin., appointed Medical Officer for the Duffield District of the Belper Union. H. Jones, L.S.A., appointed Medical Officer of Health for the Borough of Crewe. C. A. S. Ling, M.R.C.S.Eng., appointed Medical Inspector of Seamen for the Poat of Colchester. R. Morrison, L.R.C.P. and S.Edin., appointed Medical Officer for the Duffield District of the Belper Union. E. B. Osmond, L.R.C.P.Lond., M.R.C.S.Eng., appointed Honorary Consulting Medical Officer to the Pontefract Dispensary. J. Paulin, M.R.C.P., L.R.C.S.Edin., appointed Medical Officer for the Ashton-on-Mersey District of the Altrincham Union. F. J. C. Parsons, L.R.C.P.Lond., M.R.C.S. Eng., reappointed Medical Officer of Health for the Bridgwater Urban and Rural Sanitary Districts. A. Rider, M.B.Lond., M.R.C.S., L.R.C.P., appointed Assistant Surgeon to the Royal Albert Hospital, Devonport. J. D. Roberts, M.R.C.S.Eng., appointed Surgeon to the Ealing Cottage Hospital. J. F. W. Silk, M.D.Lond., M.R.C.S., L.S.A., appointed Anæsthetist and Instructor in Anæsthetics in King's College Hospital. J. H. Thompson, L.R.C.P.I., L.R.C.S.Edin., reappointed Medical Officer of Health for the Mytholmroyd Urban Sanitary District. Dr. W. A. Turner, appointed Physician to Out-patients and Registrar, Hospital for Epilepsy and Paralysis. J. J. D. Vernon, M.B.Lond., M.R.C.S.Eng., appointed Medical Officer of Health for the Audley Urban Sanitary District of the Newcastle-under-Lyme Union. F. C. Wallis, M.B.Cantab., F.R.C.S., appointed Assistant Surgeon at Charing Cross Hospital. E. Williams, L.R.C.P.I., M.R.C.S.Eng., appointed Medical Officer of Health for the Llangefni Urban Sanitary District of the Anglesey Union. T. J. Wood, M.D.Lond., M.R.C.S., L.R.C.P., appointed House Surgeon to the Bradford Infirmary. J. W. Carr, M.B., B.S.Lond., M.R.C.P., F.R.C.S., has been appointed Assistant Physician to the Royal Free Hospital.

### VACANCIES.

The following vacancies are announced this week, the amount of salary in each case being given after the name of the institution:

**Resident Medical Officers.**

Bristol Hospital for Sick Children and Women. House Surgeon; doubly qualified. Salary £100 per annum, with rooms and attendance. Applications marked "House Surgeon" to the Secretary, H. Lawford Jones, by August 12th.

Cumberland Infirmary, Carlisle. Assistant House Surgeon. Salary £40 per annum, with board, lodging, and washing. Applications and testimonials to the Secretary, by August 17th.

### The Hospital Advertiser.

### MEDICAL VACANCIES AND APPOINTMENTS—(*continued*)

Royal Infirmary, Hull. Senior House Surgeon. Unmarried. Salary 100 guineas per annum, with board and furnished apartments. Testimonials to be sent to the Chairman of the House Committee by August 15th.

Victoria Infirmary of Glasgow. Superintendent and Resident Medical Officer. Applications to Francis Bisset, Secretary, 22, Carlton Place, Glasgow, by August 15th.

**Non-Resident Medical Officer.**

Westminster Hospital, Broad Sanctuary, S.W. Surgeon. Must be Fellow of the Royal College of Surgeons of England. Applications with testimonials, to the Secretary, S. M. Quennell, by August 22nd.

### PASS LISTS.

**University of Edinburgh.**

The following gentlemen have passed in Anatomy, &c.:—Messrs. H. H. Balfour, F. A. Clarke, C. B. Crampton, D. F. Dewar, J. Forbes, M.A., B.Sc., Edmond Frost, C. B. Johnstone, H. Jones, G. Laurence, W. C. A. M'Dowell, B.A., T. T. Ormerod, E. G. Richards, T. E. E. Roddis, J. B. Stewart, W. M. Taylor, D. G. M. Teague, and P. W. Wilkinson.

The following is the official list of candidates who passed the first and second professional examinations last month:—

First Professional Examination.—F. T. H. Adamson, B. C. R. Aldren, J. Allison, E. B. Anderton, R. W. Anthony, Eustace Arkwright, F. G. Ballantyne, J. H. Bam, T. Biggam, R. B. Black, H. F. Boland, Leonard Boyer, G. M. Bucher, H. N. Bundey, B. W. Burwood, C. A. Brugman, H. N. Chambers, G. L. Chiene, T. S. Church, C. B. Clarke, E. F. Cozhlan, R. Craven, T. W. A. Daman, J. L. Davis, D. C. Dickson, J. Donaldson, J. Dorman, J. F. Falconer, G. L. Findlay, D. L. Fisher, A. W. Fletcher, J. G. Forbes, J. Forrest, J. G. Forsyth, W. H. Fowler, A. F. Fraser, J. Fraser, J. M. Fraser, G. Gatenby, A. Gibson, J. C. Hamilton, W. Hamilton (with distinction), F. S. Harper, W. G. Heath, P. T. Herring, R. P. Hill, H. O. Hobson, G. B. Hollings, Oswald Horrocks, C. H. Johnson, J. M. Johnson, A. H. Keun, Imdadali Khan, Evariste Laval, A. M. Limont, G. H. List, J. D. Lithgow, A. M. Little, J. S. Lyle, A. B. MacCarthy, A. J. M Clymont, J. D. M'Crindle, Ian L. MacInnes, J. W. M'Intosh, G. M'Kellar, T. D. M'Laren, C. C. Macmillan, J. Malcolm, Harold Malcolmson, W. Martin, T. M. Metcalfe, W. M. Milne, F. W. More, J. H. R. Morrow, W. Mowat, J. Muir, J. C. Noronha, Nigel Oliphant, M. J. O'Shea, G. H. Parry, J. K. Paterson, J. H. P. Paton, J. H. Patterson, T. H. Pemberton, E. E. Porritt, F. W. Price, A. E. Richard, J. Richards, R. G. Riddell, A. A. Robinson, Gerald Sander, J. T. Scott, G. S. Small, H. W. Smith, W. R. Somerset, J. F. Stewart, J. W. Struthers, J. P. Sturrock, M.A., H. V. Taylor, G. C. Thomas, H. M. Traquair (with distinction), T. B. Unwin, W. J. Veecock, G. A. Vincent, C. P. B. Wall, D. Wallace, D. C. Wallace, Addie White, F. M. Willcox (with distinction), J. R. Williamson, and W. M. Wilson.

Second Professional Examination.—H. de M. Alexander, G. Anderson, J. Anderson, J. W. Anderson, W. B. Anderson, A. W. M. Auden, B.A., H. Bateson, A. S. F. Birrell, A. Bremner, E. E. Brierley, C. S. Cartreil, P. F. Chapman, G. P. P. Clapham, H. M. Crosby, R. Cross, E. R. Dodds, H. Downes, E. A. W. English, H. J. Ewald, D. A. Farquharson, J. A. Featherstone, R. W. L. Fernandes, A. N. Fleming, J. S. Flett, Harvie Forrester, R. M. Freer, W. A. Gibb, A. J. Gibson, J. A. Gibson, C. F. Giddy, W. Glegg, T. A. Glover, J. O. Goldie, R. Grieve, J. H. K. Griffiths, W. Haig, S. H. Hall, R. P. Heddle, E. R. Holmes, M. Hughes, J. A. Hunter, D. M. Hutton (B.Sc.), J. H. Inman, L. V. Jacques, J. K. Jamieson, G. J. Jenkins, A. L. Jones, D. J. Jones, T. H. Jones, J. G. Kay, J. W. Keighley, J. I. Kelly, T. B. Kenny, J. Landsborough, D. Lawson, M.A., W. Leslie, H. L. Lewis, A. C. Lupton, S. F. Lusk, R. M'Camon, C. J. L. Macfadden, J. A. Macfarlane, G. W. M'Intosh, J. C. Mackenzie, W. H. Mackenzie, J. Mackinnon, G. D. M'Rae, W. Mailer, J. G. Masson, S. Messulam, A. Mitchell, C. H. D. Moore, R. C. Morris, M.A., P. J. Munn, H. V. Munster, A. G. Naylor, A. J. M. Paget, J. M. N. Paton, C. W. Peach, T. C. Penfold, C. E. Potter, W. L. Pritchard, F. G. Proudfoot, M.A., P. M. Ragg, P. Rattray, J. C. Rees, W. P. Richardson, R. J. Riseley, R. G. Robson, T. E. E. Roddis, A. Rodger, Laughlin Rose, M.A., R. A. Ross, V. A. Ross, J. Scott, R. S. Selby, P. D. Smith, S. H. Smith, W. A. Stephen (with distinction), W. L. Stevenson, T. Stuart, J. M. Taylor, C. G. Thompson, A. G. P. Thomson, M. L. M. Vandin, G. D. de Waal, J. S. H. Walker, G. Wallace, M.A., F. T. Walmsley, T. E. Watson, E. C. Watts, E. T. Whitaker, A. R. Wilson, M.A., F. Wilson, W. de W. Wishart (with distinction), J. B. Wood, and G. P. Yule.

### EXAMINATION IN EDINBURGH FOR THE TRIPLE QUALIFICATION.

PAPERS SET IN THE FIRST EXAMINATION.

Questions in Elementary Biology (five questions, of which four only are to be answered. Illustrate your answers by means of sketches).

1. What is a unicellular animal? Briefly describe Paramœcium, noting, along with the other features, the following: Trichocysts, myophan, and paranucleus.
2. State the meaning of the following terms, and give an example of each in the Animal Kingdom: Fission, Gemmation, Parthenogenesis, Alternation of Generations, and Polymorphism.
3. Name the seven parts in the enteron (alimentary canal) of the pigeon.
4. Describe the yeast-plant (Saccharomyces), and its two methods of reproduction.
5. Give in detail the minute structure of a leaf, as seen in microscopical section, and describe the venation, appendages, and attachment to the stem of the leaves in the Rose.

